# Syphilis Uveitis: A Case of Occam’s Razor, Hickam’s Dictum, and Crabtree’s Bludgeon

**DOI:** 10.7759/cureus.89329

**Published:** 2025-08-04

**Authors:** Dhruv Sethi, Obadeh Mohiddin, Cecilly Kelleher, Venkatkrish M Kasetty, Nitin Kumar, Uday R Desai, Abdualrahman Hamad

**Affiliations:** 1 Ophthalmology, Retina Group of Washington, Fairfax, USA; 2 Ophthalmology, Case Western Reserve University, Cleveland, USA; 3 Ophthalmology, Duke University, Durham, USA; 4 Ophthalmology, Henry Ford Health System, Detroit, USA

**Keywords:** maculopapular rash, neurosyphilis, syphilis uveitis, tb – tuberculosis, uveitis

## Abstract

This report discusses a case of diagnosing neurosyphilis in a non-classical presentation with confounding test results needing a deliberate and multidisciplinary diagnostic approach. A 38-year-old immunocompetent male presented with uveitis and a skin rash. Although serology was positive for syphilis (rapid plasma reagin 1:128), it was also positive for tuberculosis, and a dermatology consult identified the rash as psoriasis, creating a complex diagnostic picture. Based on a high suspicion of ocular syphilis, the patient received intravenous penicillin, which led to the complete resolution of his ocular symptoms and a serologically confirmed cure. This case of an ophthalmic masquerading disease was complicated by misleading clinical signs and a coexisting positive serology, serving as a reminder to maintain a broad differential diagnosis and be systematic in workup and management in order to optimize clinical outcomes.

## Introduction

Syphilis is a common sexually transmitted disease caused by the spirochete *Treponema pallidum,* with an estimated 10-12 million new infections each year [1]. The initial presentation can vary widely and be non-specific, depending on the duration of the disease. The stages of syphilis are classified into three types, based on the clinical manifestations [2]. Primary syphilis is the initial stage of inoculation classically with a genital chancre, secondary syphilis has bacteremia and wide dissemination of the spirochete, and late or tertiary syphilis has chronic, end-organ complications that are typically cardiovascular or neurological in nature [2]. 

Syphilis is still largely important worldwide; one reason is that it facilitates human immunodeficiency virus (HIV) transmission [3]. This is observed in epidemiological studies, which show a disproportionate number of homosexual men are afflicted, with higher rates of them also being HIV antibody positive [4].

Ocular syphilis, which is a form of neurosyphilis, typically manifests as uveitis. It can affect several parts of the eye, presenting as anterior uveitis, posterior uveitis, panuveitis, retinitis, papillitis, and scleritis [1]. Syphilitic uveitis is a rare condition accounting for 1%-2% of all uveitis [5]. The treatment varies based on the stage of the disease but usually warrants aggressive, prolonged intravenous penicillin. 

Herein, we describe an interesting case of uveitis with syphilis and tuberculous coinfection in a previously healthy patient. 

## Case presentation

**Figure 1:**
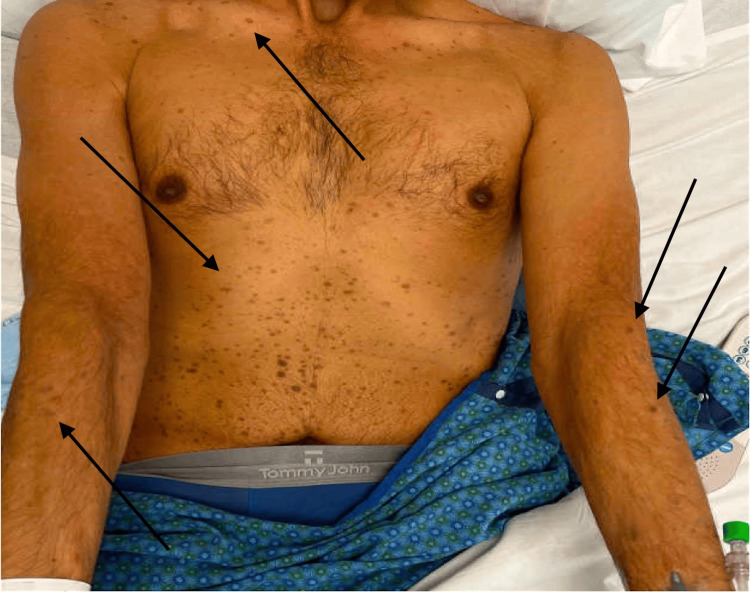
Diffuse maculopapular rash involving the forearms and chest (black arrows).

**Figure 2:**
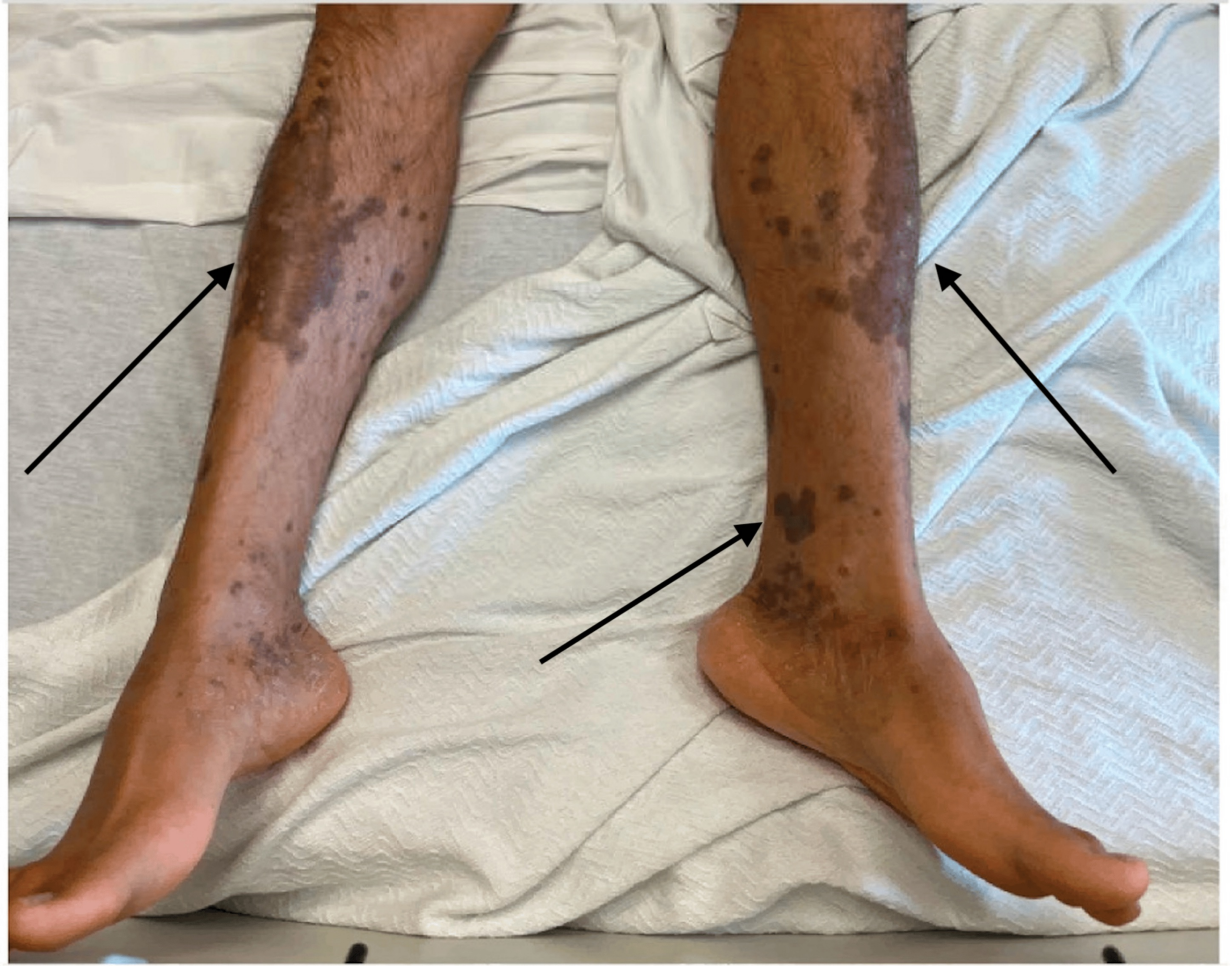
Maculopapular rash involving the lower extremities and soles (black arrows).

A 38-year-old South Asian male patient presented to his primary care physician with complaints of sore throat and painless blurry vision that was worse in the left eye than the right eye. He had difficulty seeing at night, which impaired his ability to drive. He also had a one-week history of large floaters that had decreased his visual acuity. He reported similar episodes of visual symptoms occurring sporadically over the previous three months alongside a flu-like illness. During the most recent episode, he applied warm compresses for attempted relief in addition to a trial of steroid eye drops received from a friend. He then presented to our retina service, where history taking revealed an extensive travel history to Pakistan, with bi-annual visits. Additionally, the patient reported being sexually active with both men and women, with his most recent sexual encounter occurring six months ago. He reported engaging in both oral and vaginal sex and denied any previous history of sexually transmitted infections (STIs). He has had no discussions with his previous partners about their STI history. He did not note any genital lesions but did recall a flu-like illness approximately three months ago, with the onset of the visual symptoms beginning shortly afterward. The patient also endorsed a 6-month history of a diffuse skin rash involving the palms and soles that has persisted to this visit (Figures 1, 2). 

**Figure 3:**
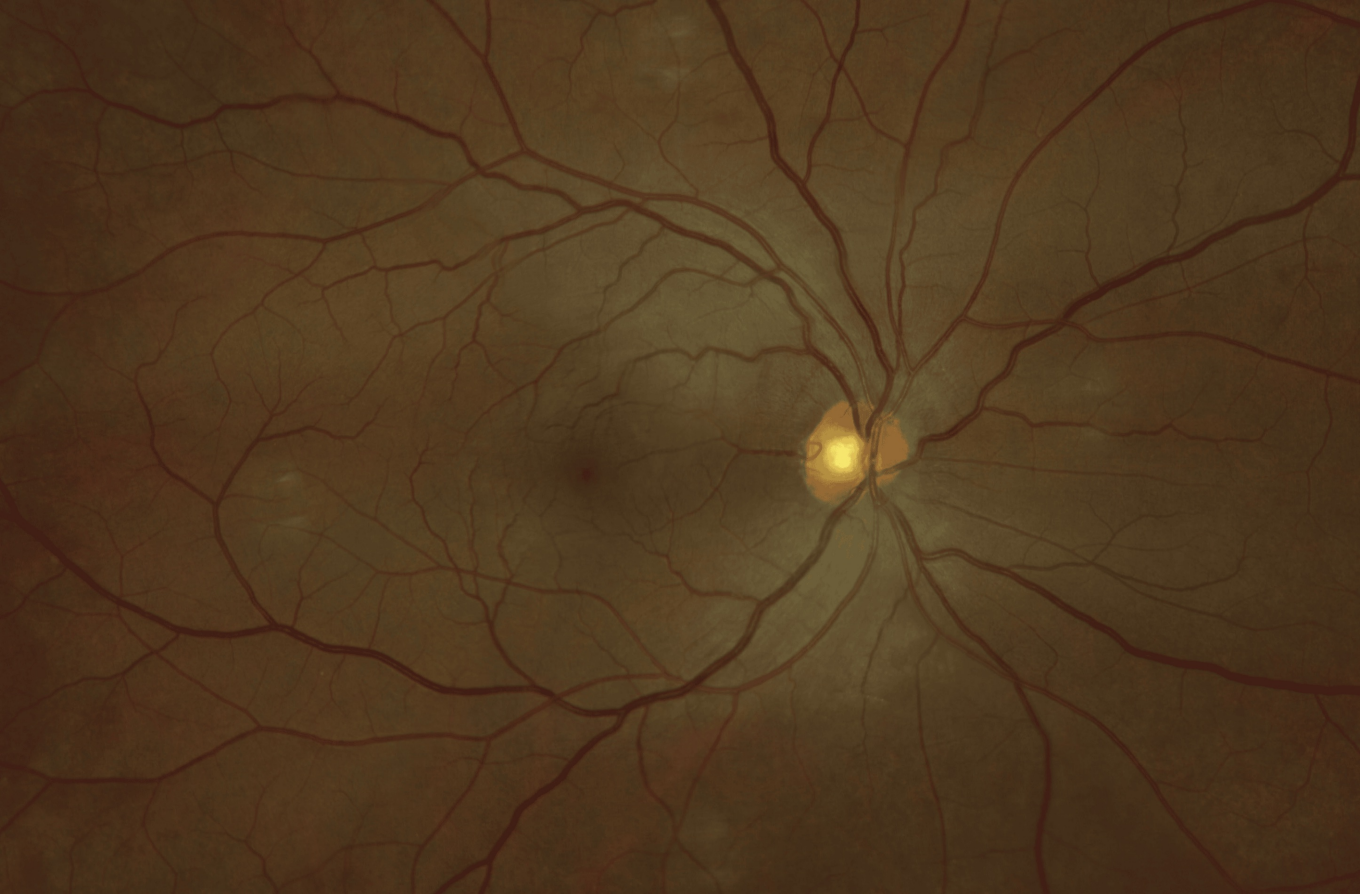
Fundus photo of the right eye, without retinal or choroidal inflammation.

**Figure 4:**
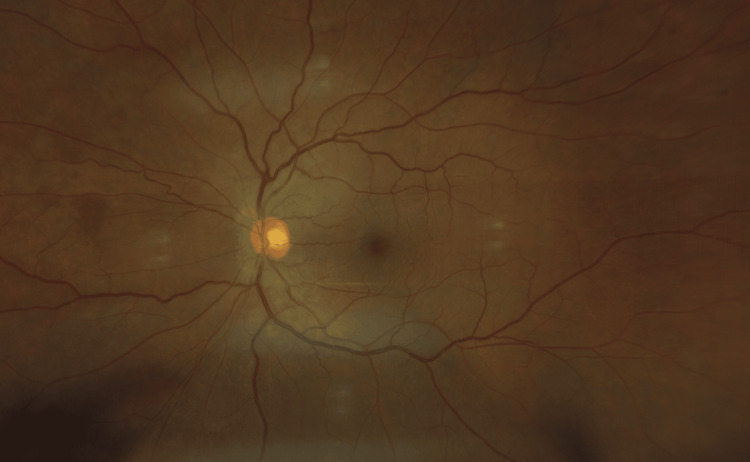
Fundus photo of the right eye, without retinal or choroidal inflammation.

**Figure 5:**
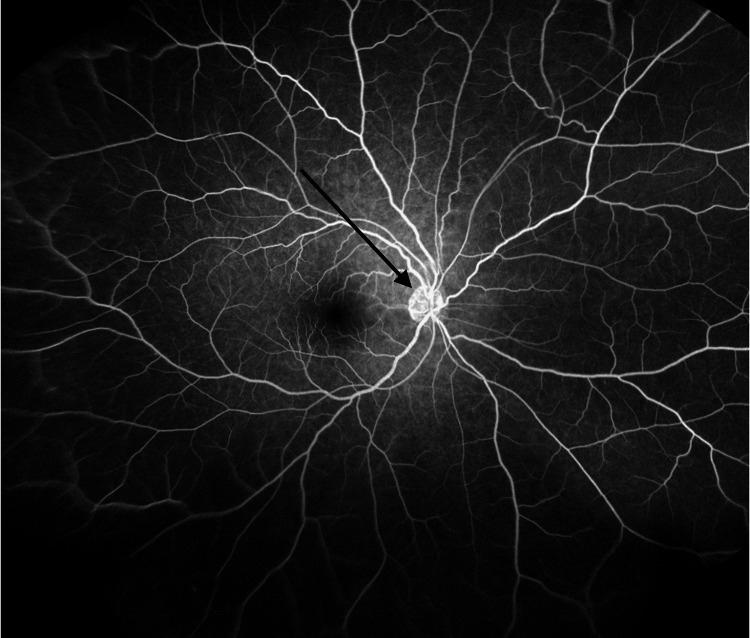
Fluorescein angiography showing mildly inflamed optic nerve of the right eye (black arrow).

**Figure 6:**
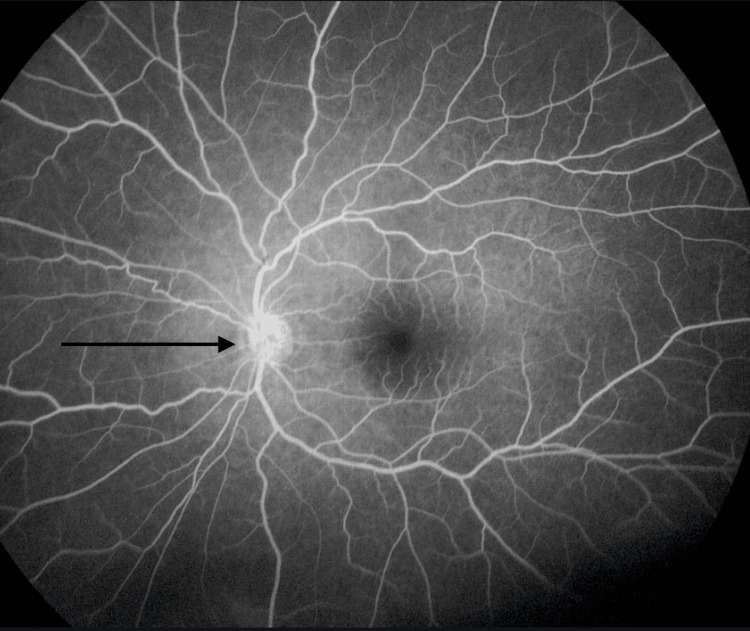
Fluorescein angiography showing mildly inflamed optic nerve of the left eye (black arrow).

**Figure 7:**
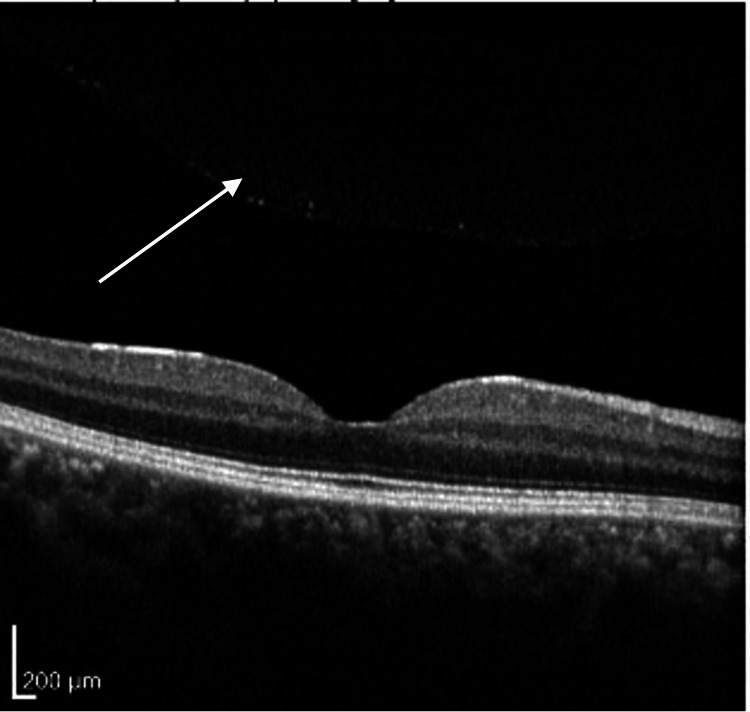
Retinal optical coherence tomography of the right macula, showing normal architecture without disruption. Inflammatory debris is visible in the superior vitreous (white arrow).

**Figure 8:**
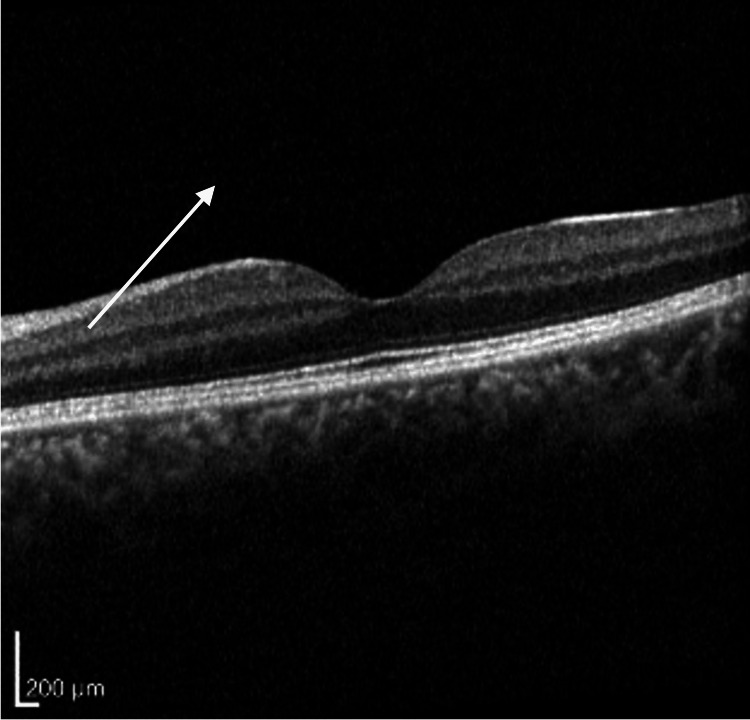
Retinal optical coherence tomography of the left macula, showing normal architecture without disruption. Vitreous inflammation is likely visible superiorly (white arrow).

On ocular examination, uncorrected visual acuity testing in the right eye was 20/20, and 20/25 in the left eye. Intraocular pressure was unremarkable in both eyes. The ocular exam revealed an anterior chamber with 0.5+ cells in the right eye and 1+ cells in the left eye as per the standardization of uveitis nomenclature. There were also trace cells (0.5+) in the anterior vitreous in both eyes. The fundus exam was unremarkable for both eyes (Figures 3, 4), while fluorescein angiography demonstrated mild optic nerve inflammation (Figures 5, 6). No posterior vitreous detachments or floaters were seen in both eyes. Optical coherence tomography (OCT) was also conducted, which suggested subtle vitritis (Figures 7, 8). 

**Figure 9:**
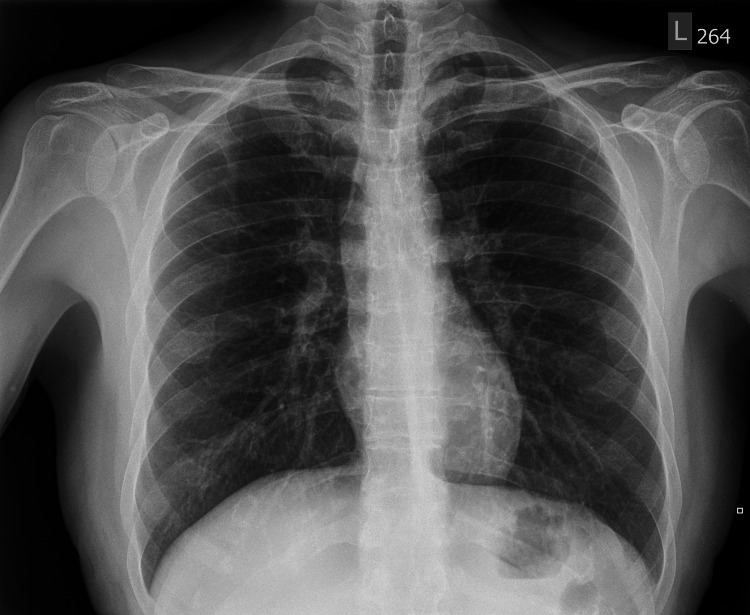
Chest X-ray without evidence of any cardiopulmonary process. No evidence of pulmonary sarcoidosis or tuberculosis.

Subsequent tests were performed to assess potential causes of uveitis, with positive QuantiFERON-tuberculosis (TB) Gold Plus assay, reactive syphilis IgM/IgG serology, 1:128 rapid plasma reagin (RPR), and angiotensin-converting enzyme being positive at 65 units/liter (reference normal defined as less than 52 units/liter). HIV, gonorrhea, and chlamydia testing was negative. Complete blood count and complete metabolic panel were largely unremarkable with no evidence of inflammation or leukocytosis. Chest X-ray revealed no evidence of active pulmonary TB or sarcoid (Figure 9). Dermatology also participated in his care, identifying the rash as diffuse, maculopapular, and involving the soles, as most consistent with a flare-up of existing psoriasis. 

Due to the results of the positive syphilis and tuberculosis testing and non-specific anterior and intermediate uveitis findings, the patient could not be differentiated as having uveitis secondary to one infectious cause over the other and was admitted to the infectious disease service. The patient began a 14-day course of intravenous penicillin inpatient and received a course of topical prednisolone acetate 1% for local inflammatory control. Due to a negative chest X-ray and a lack of respiratory TB symptoms, the TB treatment was not indicated during the acute period of hospitalization. He was eventually discharged with follow-up for assessment of the resolution of visual symptoms.

At follow-up one month after hospitalization, the patient reported clinical improvement of visual cloudiness, photosensitivity, and floaters and was measured to have excellent visual acuity. At eight months after penicillin treatment, his RPR quantitative titers showed a greater than fourfold decrease from 1:128 to 1:8, showing effective treatment of syphilis infection. The patient also reported at this visit that all vision concerns had remained resolved.

## Discussion

This case posed a diagnostic challenge due to non-specific symptoms and examination in a young, healthy, immunocompetent patient. Laboratory investigations and physical examinations showed atypical manifestations of disease with evidence of multiple possible infectious etiologies, which further clouded the ability to make a straightforward diagnosis. 

There are a few guiding philosophical principles of problem-solving that are commonly used in the practice of medicine. These aid in the development and testing of hypotheses and are used in our everyday logic and rationale by both new and experienced clinicians in order to make rational, evidence-based diagnoses and management plans. Occam’s razor is a philosophical principle that suggests that the simplest diagnosis is the most likely to be correct [6], while Hickam’s dictum is often posited as the counterargument stating that multiple disease entities are more likely than one, and that multiple diagnoses may better explain an array of symptoms [7]. Additionally, a lesser-known corollary is Crabtree’s bludgeon, in which a presenting sign or symptom may be a distraction away from the correct diagnosis [8]. All three of these philosophical principles can be applied to our case, and without acquiring a comprehensive history and review of systems, in addition to keeping the mind open to multiple differentials, there is an increased likelihood of incorrect treatment and care of patients. 

Upon evaluation in the ophthalmology clinic with a comprehensive history and review of systems, a slit lamp and fundus examination, and tests including fluorescein angiography, OCT macula, and fundus photography, anterior/intermediate uveitis was diagnosed, defined by the anatomical areas with and without inflammation. Initial testing screened for sarcoidosis, TB, and syphilis, which are commonly referred to as the great masqueraders of inflammation in ophthalmology, given that these conditions can present vaguely, with non-specific presentations that are important to rule out before initiating systemic steroids. 

Positive RPR and diffuse maculopapular rash involving the forearms and soles satisfy Occam’s razor to make the seemingly conclusive diagnosis of syphilitic uveitis. However, with the assistance of dermatology, these cutaneous findings were attributed to psoriasis, who recommended that this dermatologic manifestation should not be used to support the diagnosis of syphilis. This instance showcases Crabtree’s bludgeon, in which the initial laboratory findings and physical examination were used to fit the presumptive diagnosis, which, in this case, was coincidentally correct, but this thought process can easily lead to a falsely narrow differential and substandard care. 

​In response to the positive tuberculous serology, a chest X-ray was obtained, which showed no evidence of active tuberculosis. Hickam’s dictum allows for the potential etiology of the uveitis to be the underlying syphilis infection as well as latent tuberculosis, given that it is always possible to have multiple diseases or infections. 

## Conclusions

This case emphasizes the importance of acquiring a comprehensive history and review of symptoms in the setting of multiple infectious diseases with non-specific ocular examination findings. A multidisciplinary approach to management is of great significance to accurately diagnose and treat the underlying disease and achieve relief of symptoms. It was critical in the management of this patient to have a step-wise approach to managing inflammation and a contingency plan for the next treatment/etiology to address if the inflammation had persisted even after resolution of the syphilis infection, such as considering anti-tuberculosis treatment. 
